# Peritoneal and meningeal relapse from lung adenocarcinoma after a response to gefitinib: A case report

**DOI:** 10.3892/mco.2013.122

**Published:** 2013-05-16

**Authors:** KOICHI KURISHIMA, KUNIHIKO MIYAZAKI, TOMOHIRO TAMURA, GEN OHARA, KATSUNORI KAGOHASHI, MIO KAWAGUCHI, HIROAKI SATOH

**Affiliations:** Division of Respiratory Medicine, Mito Medical Center, University of Tsukuba, Mito, Ibaraki 310-0015, Japan

**Keywords:** carcinomatous peritonitis, carcinomatous meningitis, gefitinib, recurrence, lung adenocarcinoma

## Abstract

The prognosis of carcinomatous peritonitis and meningitis is poor since ascites and focal neurological dysfunctions usually do not improve despite the available treatment options for this devastating disease. This is a case report of peritoneal and meningeal relapse from lung adenocarcinoma following a response to gefitinib. A 72-year-old woman was diagnosed with adenocarcinoma in the upper lobe of the left lung, accompanied by a massive carcinomatous pleural effusion. Following a cisplati-based chemotherapy, gefitinib was initiated, achieving satisfactory disease control. At 8 months after gefitinib initiation, the patient developed abdominal distension, gait disturbance and visual disorder of the right eye. Adenocarcinoma cells were detected in the ascitic and pleural fluids. The findings of this case report suggest that clinicians should remain vigilant for this type of metastasis, although it is rarely encountered.

## Introduction

In lung cancer patients, metastases to the peritoneum as well as to the spinal space are rare (1–10). Carcinomatous meningitis, which attracts increasing attention as a recurrence site in patients who received treatment with epidermal growth factor receptor-tyrosine kinase inhibitors (EGFR-TKIs) (2–8), is a devastating event occurring in lung cancer patients (9–10) for which currently available treatment options are limited and the prognosis remains poor (1,9–10). This is the presentation of a case of peritoneal and meningeal relapse from lung adenocarcinoma following response to gefitinib therapy. This case report conformed to the Ethical Guidelines for Clinical Studies issued by the Ministry of Health, Labor and Welfare of Japan. Comprehensive informed consent with regard to clinical significance was obtained from the patient.

## Case report

**Figure 1.**
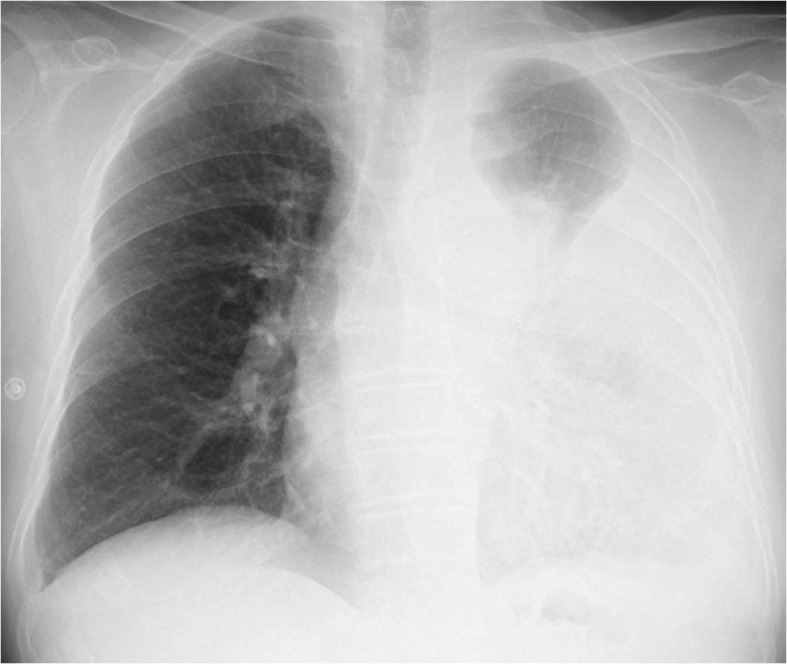
Chest radiograph at admission revealed a loculated pleural effusion in the left hemithorax.

A 72-year-old woman was admitted to our hospital complaining of a persistent cough that had appeared 2 months before. The patient had never been a smoker and had no other significant medical history. Chest radiograph at admission revealed a loculated pleural effusion in the left hemithorax (Fig. 1). The cytological diagnosis of the pleural fluid specimen was adenocarcinoma. Since there were no distant metastases or tumorous lesions in other organs where adenocarcinoma may develop, the diagnosis was lung adenocarcinoma.

**Figure 2.**
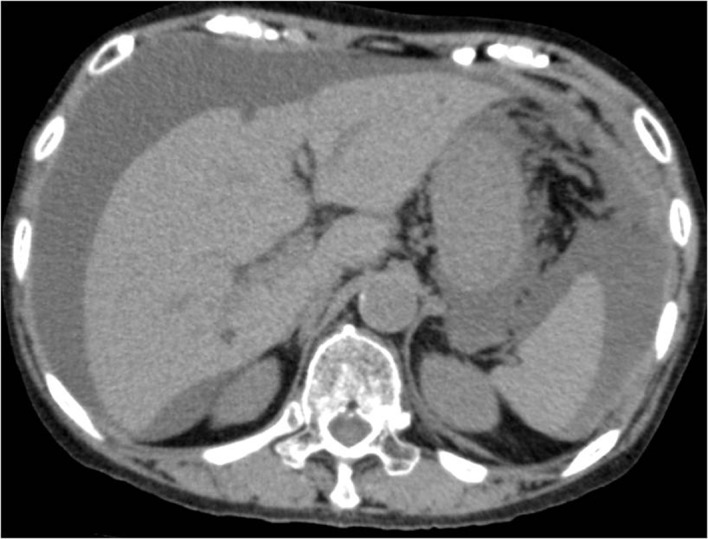
Abdominal computed tomography (CT) scan at the time of recurrence revealed massive ascites.

The patient received 4 courses of cisplatin-based chemotherapy and the response was evaluated as partial. Two months following the completion of chemotherapy, the patient developed dyspnea on exertion due to reaccumulation of left pleural fluid and the cytological pleural fluid specimens showed an EGFR mutation (L858R point mutation in exon 21). Therefore, the patient was started on 250 mg/day gefitinib therapy and the dyspnea disappeared within 2 weeks. A grade 1 (National Cancer Institute common toxicity criteria version 2.0) skin rash was observed, but it was tolerable. Eight months after the initiation of gefitinib, the patient developed abdominal distension, gait disturbance and visual disorder of the right eye. Massive ascites were revealed by abdominal computed tomography (CT) scan (Fig. 2). Positive lymph nodes were identified around the abdominal aorta, although no metastatic lesions were detected in the abdominal organs on the CT scan. Adenocarcinoma cells were detected in the ascitic and pleural fluids. Due to the deterioration of the performance status (PS 4), the patient received supportive care and succumbed to the disease 2 months following peritoneal and meningeal relapse.

## Discussion

Carcinomatous meningitis occurs in ∼5% of cancer patients (11) and is considered to be an oncological emergency requiring immediate treatment (12). Treatment options include whole-brain irradiation, systemic chemotherapy and intrathecal chemotherapy, such as methotrexate (12). However, despite these treatments, focal neurological dysfunctions do not improve in the majority of cases (13) and the prognosis remains poor, with a median survival of 2–3 months (12). Recently, the benefits of EGFR-TKIs in the treatment of carcinomatous meningitis in patients with non-small-cell lung cancer (NSCLC) have attracted attention, although all studies were case reports or case series (2–8). Furthermore, there is a high incidence of disease recurrence in the brain and leptomeninges in NSCLC patients after response to gefitinib treatment (14).

Metastatic involvement of the peritoneum is not a rare autopsy finding (15–17). Based on previous studies, the incidence of carcinomatous peritonitis is 2.7–16% in lung cancer patients (15–17). However, carcinomatous peritonitis from lung cancer is infrequently encountered and clinical reports on this type of distant metastasis are rare. We previously reported that 12 (1.2%) out of 1,041 patients with lung cancer developed carcinomatous peritonitis (1). The incidence of peritoneal carcinomatosis was high among patients with adenocarcinoma. The median survival time from the diagnosis of carcinomatous peritonitis was 2 months (1). Our previous and present findings, taken together, suggest that ascites accompanying metastasis to abdominal organs and pleural seeding in a patient with known lung cancer should be regarded as metastasis (1).

A previous study by Su *et al* (18) also reported clinicopathological findings in 30 lung cancer patients with carcinomatous peritonitis. According to their findings, the most frequent abdominal symptom of carcinomatous peritonitis was abdominal distension and the most common histological type of lung cancer was adenocarcinoma. Eighty percent of the patients developed malignant pleural effusions prior to the diagnosis of carcinomatous peritonitis. Notably, 4 patients with lung adenocarcinoma and carcinomatous peritonitis were treated with gefitinib. Two of them, who responded to therapy, demonstrated improved abdominal conditions with gradually diminishing ascites and survived for 203 and 343 days, in marked contrast to the previously reported poor median survival of 15 days (18). There is also another previous report of a patient with lung adenocarcinoma and carcinomatous peritonitis who responded to gefitinib therapy (19). It should be noted that these patients were successfully treated for carcinomatous peritonitis by gefitinib (18,19). However, there were also patients with peritoneal recurrence following successful treatment with gefitinib, such as the present case report. The existence of two different responses (good response of peritonitis to gefitinib and peritoneal recurrence following a good response to gefitinib), seems to be similar to the observations made in lung cancer patients with carcinomatous meningitis.

Irrespective of the peritoneal or subarachnoid space, there may be other uncommon recurrence sites in lung cancer patients. This suggests that such an unusual recurrence may be associated with insufficient concentration of gefitinib at these sites. Although it is rarely encountered, clinicians should remain vigilant for this type of recurrence in lung cancer patients, even following the successful treatment with EFFR-TKIs.

## References

[1] Satoh H, Ishikawa H, Yamashita YT (2001). Peritoneal carcinomatosis in lung cancer patients. Oncol Rep.

[2] Kim MK, Lee KH, Lee JK (2005). Gefitinib is also active for carcinomatous meningitis in NSCLC. Lung Cancer.

[3] Yi HG, Kim HJ, Kim YJ (2009). Epidermal growth factor receptor (EGFR) tyrosine kinase inhibitors (TKIs) are effective for leptomeningeal metastasis from non-small cell lung cancer patients with sensitive EGFR mutation or other predictive factors of good response for EGFR TKI. Lung Cancer.

[4] Langer C, Soria JC (2010). The role of anti-epidermal growth factor receptor and anti-vascular endothelial growth factor therapies in the treatment of non-small-cell lung cancer. Clin Lung Cancer.

[5] Katayama T, Shimizu J, Suda K (2009). Efficacy of erlotinib for brain and leptomeningeal metastases in patients with lung adenocarcinoma who showed initial good response to gefitinib. J Thorac Oncol.

[6] Ruppert AM, Beau-Faller M, Neuville A (2009). EGFR-TKI and lung adenocarcinoma with CNS relapse: interest of molecular follow-up. Eur Respir J.

[7] Togashi Y, Masago K, Fukudo M (2010). Cerebrospinal fluid concentration of erlotinib and its active metabolite OSI-420 in patients with central nervous system metastases of non-small cell lung cancer. J Thorac Oncol.

[8] Masuda T, Hattori N, Hamada A (2011). Erlotinib efficacy and cerebrospinal fluid concentration in patients with lung adenocarcinoma developing leptomeningeal metastases during gefitinib therapy. Cancer Chemother Pharmacol.

[9] Ano S, Satoh H, Nakazawa K (2007). Malignant meningitis secondary to lung adenocarcinoma: an unusual relapse. Intern Med.

[10] Jiménez Mateos A, Cabrera Naranjo F, González Hernández A (2011). Neoplastic meningitis. Review of a clinical series. Neurologia.

[11] Grossman SA, Krabak MJ (1999). Leptomeningeal carcinomatosis. Cancer Treat Rev.

[12] Wasserstrom WR, Glass JP, Posner JB (1982). Diagnosis and treatment of leptomeningeal metastases from solid tumors: experience with 90 patients. Cancer.

[13] Strik H, Prömmel P (2010). Diagnosis and individualized therapy of neoplastic meningitis. Expert Rev Anticancer Ther.

[14] Omuro AM, Kris MG, Miller VA (2005). High incidence of disease recurrence in the brain and leptomeninges in patients with nonsmall cell lung carcinoma after response to gefitinib. Cancer.

[15] McNeill PM, Wagman LD, Neifeld JP (1987). Small bowel metastases from primary carcinoma of the lung. Cancer.

[16] Abrams HL, Spiro R, Goldstein N (1950). Metastases in carcinoma; analysis of 1000 autopsied cases. Cancer.

[17] Warren S, Gates O (1964). Lung cancer and metastasis. Arch Pathol.

[18] Su HT, Tsai CM, Perng RP (2008). Peritoneal carcinomatosis in lung cancer. Respirology.

[19] Nakata H, Tuji T, Ohsaki Y (2004). A case of peritonitis carcinomatosa of pulmonary adenocarcinoma in which gefitinib was effective. Cancer Chemother.

